# Morphological and Molecular Characterization of *Paragonimus skrjabini* Complex from Yunnan, China: A Brief Report

**DOI:** 10.1007/s11686-021-00461-w

**Published:** 2021-08-21

**Authors:** Qiu-Hong Shu, Shu-De Li, Ming Tian, Yong Meng, Shu-Mei-Qi He, Min Zhu, Miao-Miao Wang, Wen-Lin Wang

**Affiliations:** 1 Department of Cardiology, The Second Affiliated Hospital of Kunming Medical University, No. 374, Dianmian Road, Kunming, 651010 Yunnan China; 2 School of Basic Medical Sciences, Kunming Medical University, No. 1168, Chunrong West Road, Yuhua Street, Chenggong District, Kunming, 65050 Yunnan China; 3 Department of Parasitology, Faculty of Basic Medicine, Kunming Medical University, No. 1168, Chunrong West Road, Yuhua Street, Chenggong District, Kunming, 65050 Yunnan China

**Keywords:** *P. skrjabini*, Phylogenetic analysis, Paragonimiasis

## Abstract

**Purpose:**

To perform environmental sampling and molecular identification of *Paragonimus* in endemic regions, which may help in minimizing transmission among humans.

**Methods:**

Mountain crabs from the genus *Potamiscus* were collected and the encysted metacercariae were extracted and subjected to morphological identification, followed by animal inoculation in Sprague–Dawley (SD) rats. After 112 days of infection, animals were killed and adult worms were extracted from lungs and muscles. The morphology of adult worms was characterized by microscopy and molecular identification was done by polymerase chain reaction, followed by sequencing of *cox1* and *ITS2* genes. Phylogenetic analysis was done by maximum parsimony method.

**Results:**

A total of 447 crabs were captured from the streams of Tongchang Town, Jinping County, Yunnan Province, China. The infection rate was found to be 41% (186 out of 447 crabs). The metacercariae of *Paragonimus skrjabini* was identified by the characteristics round or spherical encysted form measuring 410 to 460 × 400 to 460 µm. After animal infection in SD rats, adults were presumptively confirmed to be *P. skrjabini*, which was also confirmed by gene amplification and sequence analysis of *cox1* and *ITS2* regions. *Paragonimus skrjabini* clustered with previously reported *P. skrjabini* from Yunnan and Vietnam. The confidence values of their branches were > 95%. Phylogenetic analysis of the *ITS2* region revealed two distinct clusters with distinct geographical grouping. Phylogenetic analysis with the combined data sets reiterated the geographical grouping with *P. skrjabini* from Yunnan clustering with strains from Vietnam.

**Conclusion:**

Metacercariae of *P. skrjabini* was discovered in freshwater crabs in Yunnan province, China, and the strains were phylogenetically related to *P. skrjabini* from Vietnam.

## Introduction

Paragonimiasis is an important food-borne zoonosis, especially in Asian countries. This parasitic infection manifests as acute or chronic lung infection caused by the trematodes of the genus *Paragonimus*. The genus *Paragonimus* consists of varied species such as *Paragonimus skrjabini* complex and the *Paragonimus ohirai* complex, overlapping the geographical ranges of the *Paragonimus westermani* complex [1]. The morphological features of large metacercariae found for the first time in Vietnam resembled that of *Paragonimus skrjabini* [2]. In Asia, *P. skrjabini* is an important pathogen, along with *P. westermani*, causing infections in humans throughout Asia. Among the different species complexes, *P. skrjabini* has been previously reported mainly in East Asia and China [3]. Initially thought to be a pathogen exclusively found in China, it has recently been reported from Japan, India, and Vietnam [4].

The life cycle of *Paragonimus* is relatively complex that requires a minimum of three hosts including a definitive host and two intermediate hosts [5]. The first and second intermediate hosts are frequently snails belonging to the families Assimineidae and Hydrobiidae and crabs belonging to the families Potamidae and Parathelphusidae [6]. Different species of *Paragonimus* have their own predilection for infecting specific genera of snails and crabs and hence the epidemiological prevalence of different species of *Paragonimus* is determined by the existence of suitable hosts. Some species of *Paragonimus* have been identified only from intermediate hosts in certain geographies that suggest infection and maintenance in non-human mammals [7, 8]. In Sichuan province, 18 species of freshwater crabs such as *Stigmatomma. denticulatum*, *Sinopotamon. yaanense*, *Sinopotamon. davidi*, and *Haberma nanum* were found to act as the second intermediate host [9].

The disease burden of paragonimiasis in China is the highest among all the countries due to dietary patterns. Nearly 80% of the *Paragonimus* species reported worldwide originated from China, which also has the highest disease burden in the world with 7.83% of prevalence of human paragonimiasis [10]. Also, China is considered as an ecological hotspot with high species diversity and endemicity of the snail and crab intermediate hosts of *Paragonimus* spp. Considering the vast landscape of China and the varied biotopes, the spread of specific species of *Paragonimus* is also presumed to be distinct in different provinces [10]. Yunnan is a province in southern part of China, which is more proximal to south-east Asian countries where *P. heterotremus* is the most prevalent causative organism of human paragonimiasis [6]. Although the incidence of paragonimiasis is high among the Chinese population, studies reporting the morphological and molecular characterization on *P. skrjabini* causing paragonimiasis are limited. In addition, the morphological characterization of *Paragonimus* species from non-mammalian hosts will help to understand the genetic variation and also to suggest suitable lifestyle modifications for populations at risk of paragonimiasis.

Here, we report the discovery of *P. skrjabini* metacercariae from mountain crabs from Yunnan Province, China. Adult worms were obtained by experimental infection of the metacercariae in experimental rats. The morphological and molecular relationships with other geographical populations in the *P. skrjabini* species complex are discussed further.

## Materials and Methods

### Parasitological Methods

**Fig. 1:**
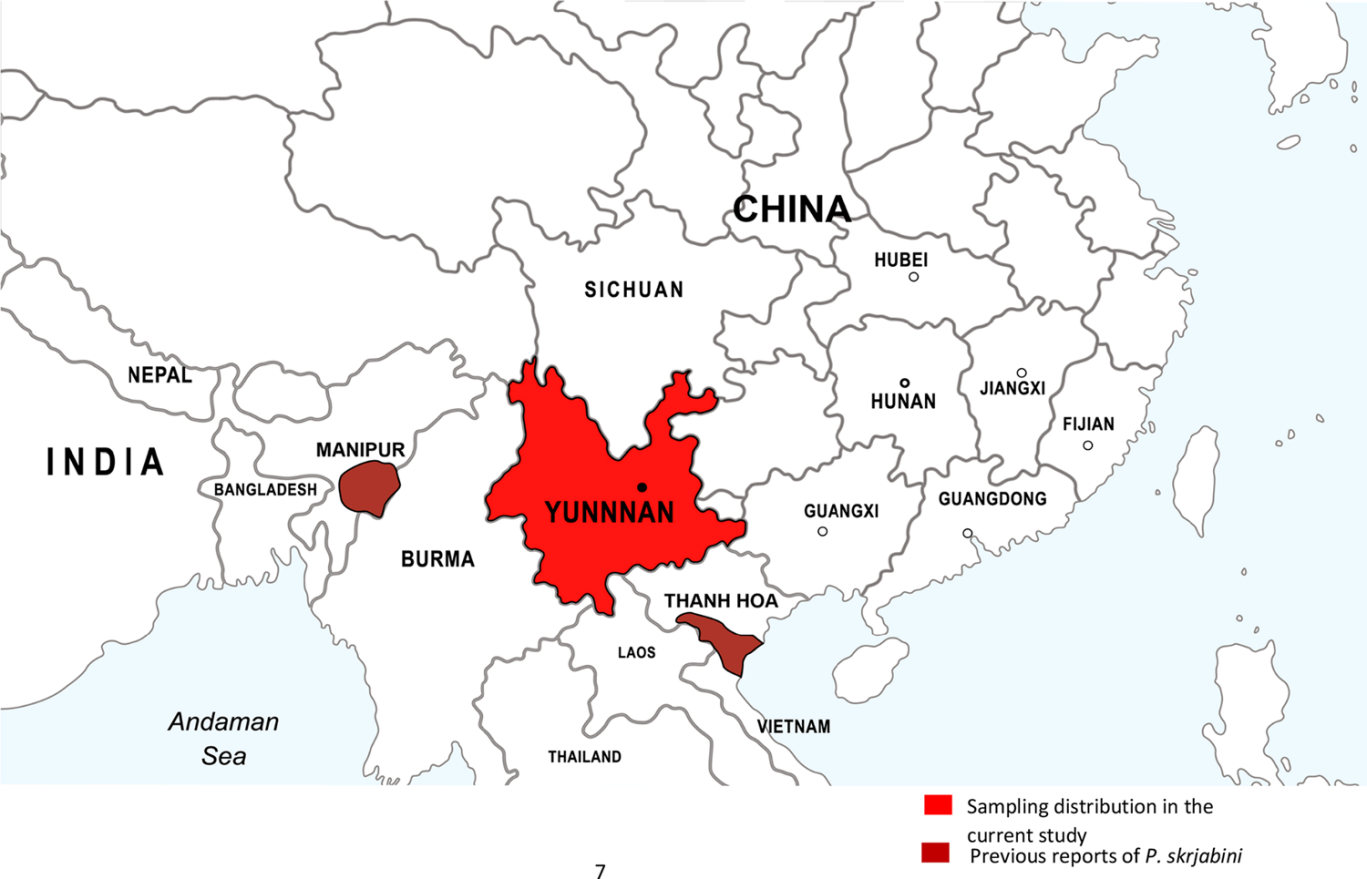
Sampling distribution and previous reports of *P. skrjabini*

The *Paragonimus* metacercariae were isolated from naturally infected mountain crabs with male crabs having hair on all eight legs and the presence of pointed umbilicus, belonging to the genus *Potamiscus*, the second intermediate hosts from Tongchang Town, Jinping County, Yunnan Province, China (Fig. 1). The sampling locations are mainly the moving streams of water without impeding vegetation. The morphological identification of the secondary hosts was done according to the classification method of “Chinese Medical Crustaceans” [11].

The crabs were crushed in a mortar, followed by sieving and washed with distilled water into a sedimentation cup with distilled water. The filtration was done with a filter of pore size 200 µm and 1000 µm. The supernatant was discarded after 20 min and the same step was repeated four to five times. The sediment was then placed in a glass dish for microscopic biological observation. The metacercariae of *Paragonimus* were counted under the microscope and a part of the sediment with the metacercariae were fixed with absolute ethanol and stored in a refrigerator at 4 °C for molecular biological experiments.

### Experimental Infection

Freshly isolated live metacercariae of *Paragonimus* (presumptively identified as *P. skrjabini* based on morphology) [12] were then injected intraperitoneally (15 metacercariae per rat) into paragonimiasis-negative SD rats (purchased from the Laboratory Animal Department of Kunming Medical University). All the animals were handled in accordance with the Guide for the Care and Use of Laboratory Animals published by the US National Institutes of Health (NIH Publication No. 8523, revised 1985). All experimental protocols were approved by the Animal Care and Use Committee of Kunming Medical University (reference: KMMU2015002). Experimental animal inoculation by subcutaneous injection was done, following which, five SD rats were killed on the 2nd, 4th, and 6th week to confirm the infections of rats with adult *Paragonimus* spp. After 114 days of injection, the SD rats were dissected to isolate the cysts, eggs, and adult worms from the muscles, abdomen, liver, thoracic cavity, and lungs of SD rats. The isolated adult worms were used for genomic DNA extraction and for preparing permanent slides for microscopic confirmation by fixing them on to glass slides with alcohol, formalin, and acetic acid.

### Morphologic Identification

The morphologic features of the cysts, metacercariae, and adults were used for the presumptive identification of *P. skrjabini*. The microscopic and morphological characterization was based on the arrangement of spines on the epidermis of the adult worms, the degree of branching of the ovaries and testes, the aspect ratio and relative size of the suction discs, the characteristics of the cyst walls (presence or absence, number and thickness), the relative diameter of the suction discs, the anterior extent of the excreted bladder, the length of the probe on the mouth suction disc, the presence of pigmented granules in the body, the number of flame cells, body spine and the alignment of the nipples around the discs [13].

### Molecular Analysis

Genomic DNA was extracted from both the adult worms from SD rats and also from the metacercariae from crabs using QIAamp DNA Mini kit (QIAGEN, Hilden, Germany). The whole process was carried out in strict accordance with the instructions of the manufacturer. The final elution of DNA was done with 100 µL of distilled water. The extracted total genomic DNA was quantified and stored in the refrigerator at − 20 °C until further use.

### Polymerase Chain Reaction Amplification of *cox1 *and *ITS2*

Polymerase chain reaction (PCR) was performed with primers targeting a fragment of the partial mitochondrial cytochrome oxidase subunit 1 (*cox1*) gene and the nuclear ribosomal second internal transcribed spacer (*ITS2*) region synthesized by Shanghai Bioengineering Co., Ltd. The primers used for amplifying *cox1* gene fragments were COIF—5′-GAGGTGTATGTCCTGATTTTGCC-3′ and COIR—5′-GACCTCACCCAATGACCCTGCAACA-3′ and the primers for amplifying *ITS2* gene fragments were ITS2F—5′-GGGTACCGGATCACTCGCTCGGTG-3′ and ITS2R—5′-GGGGATCCTGGTTGCCTTAGTCTCCGC-3′ [14]. PCR was performed in 25 µL volume with 2 µL of template DNA corresponding to 0.1 ng and 1 µL of primers (10 µmol/µL), 2.5 µL of 10 × PCR buffer, 1 µL of 10 mM deoxynucleotide triphosphates (dNTP), 0.1 µL (0.5 units) of Taq enzyme (5 U/µL) and 17.4µL of PCR grade water. The PCR amplification was set up in an ice bath and nducted in the TaKaRa PCR instrument (Bao Biology Co., Ltd.) and the amplification conditions were as follows: initial denaturation of 95 °C for 3 min, followed by 35 cycles of denaturation at 93 °C for 1 min, annealing at 48 °C (for *cox1*)/60 °C (for *ITS2*) for 1 min and extension at 72 °C for 1 min, followed by final extension at 72 °C for 5 min. The expected length of the PCR fragments was 500–750 base pairs. The detection of PCR-amplified products was done by agarose gel electrophor–esis with 1.5% agarose gel immersed in 1.0% Tris–ACETATEEDTA buffer stained with ethidium bromide. The purity and quantity were estimated using the gel documentation system imaging technique (Bio-Rad Laboratories).

The PCR products were then subjected to bidirectional sequencing using the same PCR primers by Shanghai Biotechnology Co., Ltd (Hitachi fluorescent DNA sequencer SQ-3000). The forward and reverse sequences were then manually curated and aligned with the DNASTAR v7.1 software and the consensus sequence were used for bio-informatic analysis.

The initial quality check of the sequences was done by checking the coverage and alignment with previously submitted sequences in National Center for Biotechnology Information (NCBI) using the BLAST tool. Previously submitted sequences of *cox1* and *ITS2* were retrieved from NCBI and compared with the sequences obtained in this study with ClustalX software with default parameters. A phylogenetic analysis was done by the maximum parsimony (MP) method as per the Kimura 2-parameter model. Neighbor-joining method (NJ) was used for the ML heuristic method with bootstrap replicates of 1000. The analysis was performed with MEGA5.0 software [15]. The cutoff value for consensus tree was set to 75%.

## Results

### Morphological Identification

#### Metacercariae and Adult Worms

A total of 447 crabs were captured from the streams of Tongchang Town, Jinping County, Yunnan Province, China, and were included for this study. The infection rate was found to be 41% (186 out of 447 crabs). Out of the 186 crabs, a total of 551 metacercariae were isolated and identified (range: 1 to 100, mean: 2.96 per crab).

The metacercariae of *P. skrjabini* were identified by the characteristic round or spherical encysted form measuring 410 to 460 × 400 to 460 µm. The encysted form had three layers (cyst walls) with an outermost thin layer, which could be easily ruptured, a thicker middle layer and a thinner inner layer. The average thickness of the outer and inner wall was found to be 4 and 12 µm, respectively. The encysted metacercariae were excysted by mechanical shearing and the excysted metacercariae measured 650 to 670 × 370 to 420 µm. The oral and ventral suckers were 80–120 µm and 120–60 µm, in diameter, respectively. These features roughly correlated with *P. skrjabini* and the metacercariae were subjected to animal inoculation. After experimental infections, two to four adult worms were retrieved from each animal from the lungs and muscles. The dimensions of the adult worms ranged from 950 to 1250 µm × 400 to 500 µm with an oral sucker measuring 460 to 530 µm × 625 to 710 µm. The diameter of the ventral sucker ranged from 700 to 800 µm. The other characteristic features observed were in the left (660 to 2000 × 320 to 1120) and right testis (1600 to 1650 µm × 885 to 1175 µm) and an ovary (1575 to 1800 µm × 1325 to 1500 µm). Based on these morphologic features, worms that were presumptively identified as *P. skrjabini* were subjected to molecular identification.

### Molecular Identification

PCR amplification of the *cox1* and *ITS2* regions, followed by agarose gel electrophoresis, revealed amplicons of about 500 and 750 bp in length, respectively. BLAST searches using our sequences as queries found matches with 100% coverage in GenBank.

### Phylogenetic Analysis

Based on sequence similarity, a total of 8 *P. skrjabini* and the related *P. miyazakii* sequences were downloaded from NCBI and used for phylogenetic analysis (Table 1).

**Table 1:** DNA sequences of *Paragonimus spp.* used for the phylogenetic analysis

Serial number (No.)	Generic name (generic name)	Species name (specific name)	GenBank serial number (accession number)		Sampling point (location of sample)	Specimen number (sample code)*
			*cox1*	*ITS2*		
1	*Paragonimus*	*miyazakii*	AY618807	AY618757	Japan (Japan)	
2	*Paragonimus*	*miyazakii*	AY618834	AY618742	Fujian, China (Fujian Province, China)	
3	*Paragonimus*	*skrjabini*	AB703456	AB703448	Vietnam	
4	*Paragonimus*	*skrjabini*	AY618759	AY618729	Guangxi, China (Guangxi Province, China)	
5	*Paragonimus*	*skrjabini*	AY618760	AY618743	Sichuan, China (Sichuan Province, China)	
6	*Paragonimus*	*skrjabini*	AY618763	AY618748	Hubei, China (Hubei Province, China)	
7	*Paragonimus*	*skrjabini*	AY618801	AY618730	Guangdong, China (Guangdong Province, China)	
8	*Paragonimus*	*skrjabini*	AY618805	AY618734	Yunnan-China (Yunnan Province, China)	
9	*Paragonimus*	*skrjabini*	MN650827.1	–	Yunnan, China (Yunnan Province, China)	Sample28

*Sequences obtained in the study

**Fig. 2:**
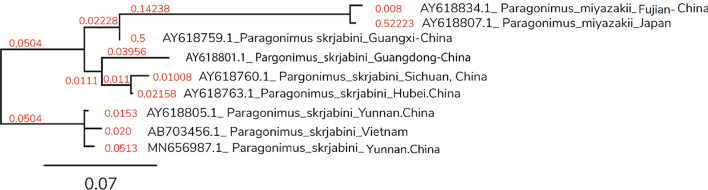
Phylogenetic tree by maximum parsimony method for COI gene

**Fig. 3:**
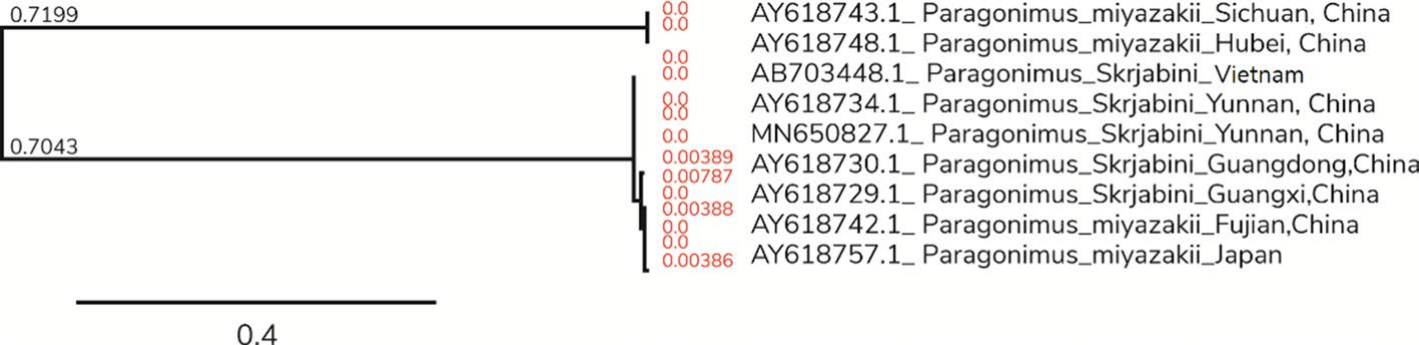
Phylogenetic tree by maximum parsimony method for *ITS2* gene

**Fig. 4:**
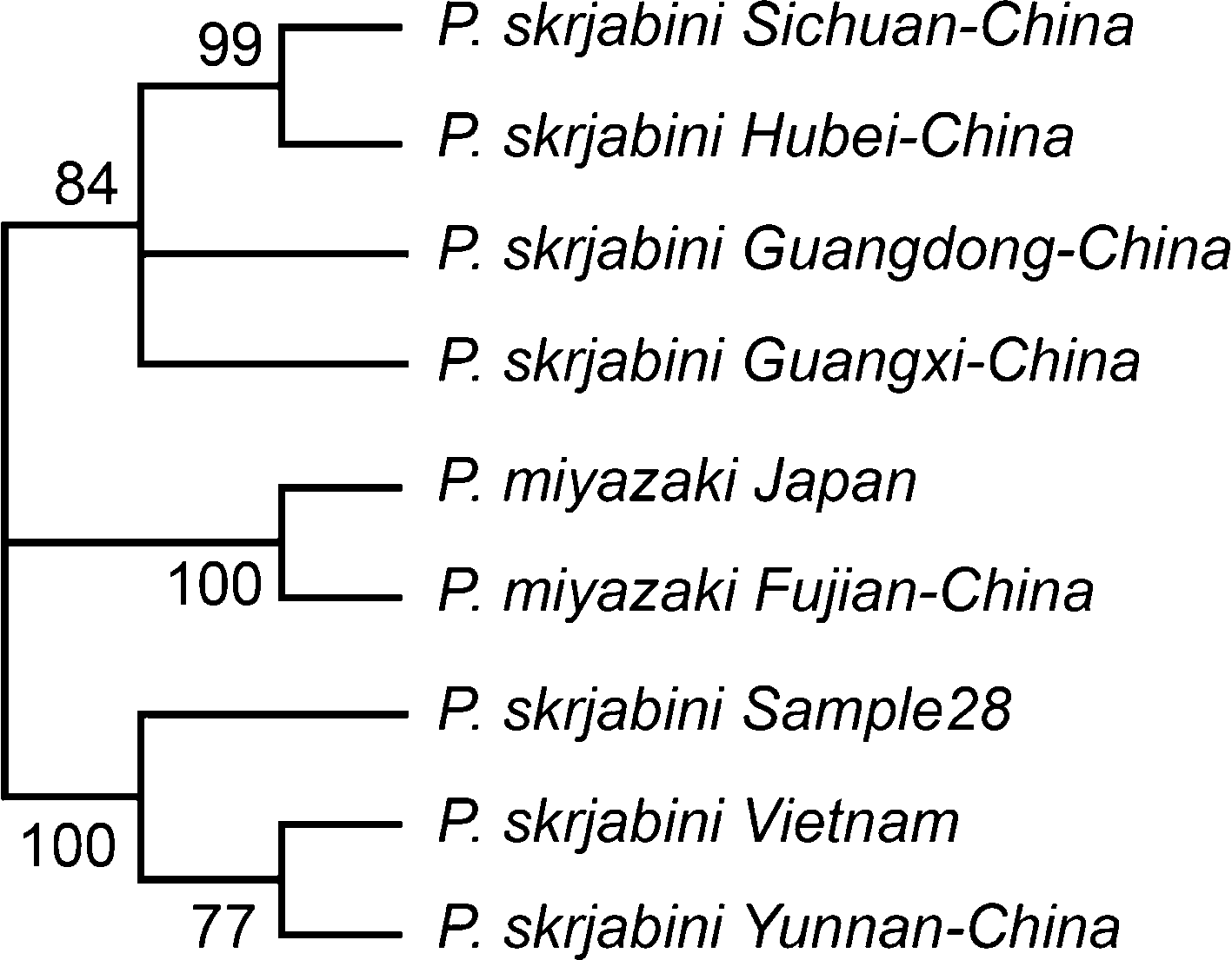
Phylogenetic tree by maximum parsimony method with both COI and *ITS2* regions

In the phylogenetic tree of COI gene (Fig. 2), *P. skrjabini* clustered with previously reported *P. skrjabini* from Yunnan and Vietnam. The confidence values of their branches were > 95% (Fig. 2). Phylogenetic analysis of the *ITS2* region (Fig. 3) revealed two distinct clusters with distinct geographical grouping. Phylogenetic analysis with the combined datasets (Fig. 4) reiterated the geographical grouping with *P. skrjabini* from Yunnan clustering with strains from Vietnam.

## Discussion

China is endemically diverse with respect to *Paragonimus* species, wherein 38 species of *Paragonimus* have been reported so far [10, 16]. Considering the diverse landscape and availability of suitable hosts, a stringent geographic predilection for occurrence is presumed in China [8]. In this study, we assessed the occurrence of a medically important species of the genus *Paragonimus*, *P. skrjabini*, and evaluated the phylogenetic relationship to substantiate the geographical predilection for occurrence.

Often, the differential morphological features of metacercariae are not well marked; hence, metacercariae stage from the secondary intermediate hosts [6] and adults from the mammalian hosts are used for morphological identification. In this study, morphological identification made at the metacercariae and adult stages were concordant with each other, further substantiating the role of morphological identification. The morphological features of the metacercariae and adult worms in the present study resembled that of *P. skrjabini*, where the adult worm was initially described from the palm civet *Paguma larvata* in Guangzhou City, Guangdong Province, China, and subsequently infections in humans were also reported from the same province [17, 18]. Previously, morphological identification-based reporting was published from Manipur, India, by Singh *et al.*, but molecular identification was not performed [4].

The infection rate by *P. skrjabini* in our study was attributed to 41% (186 out of 447 crabs). Similarly, in a study conducted in Sichuan province, the average rates of freshwater crab infection by metacercariae of *P. skrjabini* were 81% (*S. denticulatum*, Nantong), 62.5% (*S. davidi*, Shizhu), 63.2% (*S. nanum*, Fengjie), and 88.5% (*S. davidi*, Xingwen), respectively [9]. A study of *P. skrjabini* in Three Gorges Reservoir Region showed that the rate of freshwater crab infections with *P. skrjabini* metacercariae was 39.65% (1053/2656).

*ITS2* and *cox1* sequences have been widely used in intraspecific variation study in *Paragonimus* species [19–21]. The phylogenetic analysis utilizing the sequence variation in the *ITS2* and *cox1* genes revealed interesting phylogenetic features. While *cox1* phylogenetic analysis revealed distinct clustering of the related subspecies, *P. miyazakii*. In the ITS phylogeny, *P. miyazakii* clustered with *P. skrjabini* isolated from Sichuan, Fujian, Hubei, and Guangxi. *P. miyazakii* is predominantly reported from Japan, whereas *P. skrjabini* is predominantly reported from south-east Asia including Vietnam. Fujian, Hubei, and Sichuan provinces are geographically inclined towards east Asia. While Yunnan is proximal to the other South-east Asian countries. Further, in a previous study on *P. skrjabini* from Vietnam, the majority of the strains clustered with those found in Yunnan province, while *P. miyazakii* clustered with *P. skrjabini* from Fujian [22]. The phylogenetic similarities of *P. skrjabini* from Yunnan and Vietnam were also substantiated by the phylogenies derived from the combined dataset wherein strains from Yunnan and Vietnam clustered together. Notably, *P. skrjabini* species from Vietnamese and India population, together with the Chinese Yunnan population, form a distinct clade within the *P. skrjabini* complex. Further, a study by Blair *et al.* reported that *cox1* gene sequences from *P. skrjabini* and *P. miyazakii* revealed a number of differences, whereas *ITS2* gene sequences were identical between *P. skrjabini* and *P. miyazakii*. We speculate that *ITS2* gene is useful for the identification of *Paragonimus* strains, while *cox1* gene is more suitable for *Paragonimus* strains typing [23].

In conclusion, we discovered metacercariae of *P. skrjabini* in freshwater crabs in Yunnan province, China, and the strains were phylogenetically related to *P. skrjabini* from Vietnam, revealing a possible route of spread.
